# The evolution of immunohematology in South Asian countries

**DOI:** 10.4103/0973-6247.53881

**Published:** 2009-07

**Authors:** Graeme Woodfield

**Affiliations:** *Department of Molecular Medicine and Pathology, The University of Auckland, Private Bag – 92019, Auckland, New Zealand*

**Keywords:** Immunohematology, blood donors, blood donation

## Abstract

Many factors have resulted in the slow development of transfusion services in some South Asian countries. Despite difficulties, there have been some excellent developments and the outlook for the future is very positive. The biggest problems relate to the availability of the truly voluntary altruistic blood donors and considerable work is still needed to upgrade this aspect of the work. Screening for transfusion transmissible diseases is now widespread although there is still a requirement to enhance quality assurance procedures and to improve statistical definitions and collection. Other factors that have affected the evolution of immunohematology are outlined and there is now optimism for the future.

When asked to write this article my jaw dropped! How could one address such a large subject and do it justice? When I first visited some countries in South Asia in the early 1970s the problems of blood transfusion and immunohematology seemed to be insurmountable. How the situation has changed! Now it is certainly not all doom and gloom and we can be quietly optimistic about the future of transfusion medicine in the region. I personally have seen only a very small part of these developments and thus this article is biased by my own limited experience and observations.

Of course, some countries with expanding economies have been able to allocate substantial funds to the development of their blood services. As a general rule, although this is by no means absolute, the fate of the blood services is tied up with the economy of a country and the stage of development of its health program. However, it is also possible for a country with a lower gross national product to develop an adequate blood service if it has the ear of the key politicians, coupled with some visionary medical leadership in transfusion, a national blood service plan and some government funding.

Apart from finance there are some other factors that have been able to hasten the evolution of aspects of transfusion medicine and science in some countries.

One is communication, not only from international bodies but also between countries and governments. Sometimes this communication has been by conferences and meetings where different countries have been able to compare their progress and exchange ideas, often under the aegis of organizations such as the World Health Organization (WHO) and the Red Cross Society. Good examples are the Asian and Pacific regional seminars on Red Cross blood programs organized by the League of Red Cross and Red Crescent Societies in conjunction with local societies.[1]

Another has been the challenge of transfusion transmissible diseases that has forced governments to act quickly to protect their populations. HIV/AIDS has been a stimulus to safeguard the blood supply and both national and international funds have been found to upgrade laboratory services and techniques. Often the serology services introduced have been quite sophisticated and in advance of other areas of a transfusion service. Perhaps to a lesser extent, the problems of hepatitis B and C have also acted to direct attention to blood centers and their needs. Overall, very substantial progress has been made to protect blood supplies of countries in South Asia. HIV/AIDS has been a scourge in so many countries that it has meant that most governments in South Asia have been forced to release finances to ensure any collected blood is tested for this infection. Testing is now widespread and although the quality of this varies, the situation has been greatly improved. Most South Asia countries are regularly testing the majority of blood units for HIV, and Hepatitis B and C markers, although some are still not screening for the latter infection. It can be expected that national and international pressure will continue to improve the standards of blood donor infectious disease screening. Interestingly, with the emphasis on improving testing for transmissible disease, there has been far less effort to upgrade immunohematological tests to ensure serological safety. Relatively few South Asian countries screen for blood group antibodies and serological techniques that are used are often outdated, commonly relying on old-fashioned slide methods. On the positive side, more countries are utilizing the newer and simpler to use gel techniques with good results. But much remains to be done, as serological safety of blood is still very important. Even India has far to go in this direction although recent advanced training workshops are a good start.[2]

Another has been overall medical progress whereby the need for a safe and adequate supply of blood has become much more apparent and necessary, such as in heart surgery and blood diseases. Blood component usage has been much more common, requiring funding, the introduction of new equipment, and now refrigerated centrifuges and pheresis equipment is present in many more blood centers. This evolution has been quite rapid assisted by the relatively easy availability of user friendly and reliable equipment.

Some countries have benefited from imported technical and medical expertise and specific advice from better-developed blood services. Such a method can speed progress if both giver and receiver countries listen to each other. A thoughtful co-operation between the consultant and transfusion staff, without a paternalistic attitude, can achieve wonders for a blood service if staffs are prepared to really listen and act on advice given. Consultants, while sometimes expensive, can provide expert and mature advice quickly and save a great deal of time and expenditure. One problem that has emerged is the reluctance of some countries to ask for help, sometimes because of a sense of national pride. Personally, I believe that hands-on technical seminars held in the receiving country are invaluable particularly when they utilize overseas experts to provide leadership and direction. Nevertheless, small numbers of national technical and medical staff from developing countries do benefit from overseas exposure on a longer-term basis with the aim of teaching them to train local staff on their return.

Then there is the internet and email communication where the whole world has become one, and knowledge is not the exclusive preserve of a specific individual or group and answers to problems in transfusion can be obtained quickly and painlessly. A great deal of help is available using this technology and many blood services in South Asia have availed themselves of this, often free, information. The attraction of computers is great and sometimes blood services have invested too great a proportion of their funds in this activity to the detriment of other core activities.

In South Asia there has also been a trend for consolidation of smaller blood services into larger or even regional blood centers not only for efficiency but also because of costs. Although individual hospitals often have a great desire to have their own blood transfusion service, based in their hospital, this is inefficient in terms of finance and personnel as only a larger service commonly based outside the hospital environment can introduce all the modern requirements of a modern blood transfusion organization. Where the service is run by an organization such as the Red Cross, there is produced an arrangement where the transfusion service can work in conjunction with various hospitals and meet their blood needs. Models of this kind have emerged in South Asia and could be expanded. Other similar models have also emerged with new financial partnerships between governments, hospitals and the voluntary sector. Certainly there is a ‘critical mass’ of transfusion activity that is necessary for transfusion center efficiency and multiple small transfusion centers do not produce high standards of performance. This is a problem facing India and some other countries at the present time.

All these factors have meant that the improvements in blood service have been accelerated in many South Asian countries. But there is still a long way to go.

One of the things that many countries forget as they struggle to develop their blood services is that even developed countries have had to slowly evolve their services, which did not simply happen overnight. Transfusion science and medicine is a relatively new specialty; certainly my father would have had little concept of transfusion. Paid donors were used for many years in many Western countries. The concept of national blood services took time to evolve and the arguments for and against such coordinated services can still be remembered. The fight to obtain financing from less than sympathetic governmental departments was one that had to be fought by motivated medical officers who could see the future clearly. So what I am saying is that this evolution took time, we made many mistakes, and countries of South Asia need to remember that the march towards a fully fledged and efficient service may be slow and difficult. But South Asian countries now have the benefit of being able to learn from both the good and bad experiences of developed countries now that the flow of accurate and proven data is so easily available.

I have been fortunate enough to see aspects of transfusion at a time when very little in the way of organization had developed. Indeed, when I trained, blood was collected in bottles, I sharpened needles, put together blood taking sets, and sometimes washed used bottles after the transfusion of blood. We made our own blood grouping anti-sera, even our antihuman globulin, many of our tests were performed on slides and any glass tubes used in the laboratory had to be washed. There were no computers, no barcodes, but a great deal of paper documentation. Young house doctors were expected to perform the emergency grouping and cross matching prior to a transfusion, as trained competent technical staff was not always available. There were also many unexplained transfusion reactions that puzzled us at the time. We did not know of the benefits of blood group antibody screening and the management of many clinical situations was by today’s standards primitive. Donor screening was basic, with few questions asked outside the general health of the voluntary donor. Collected blood was tested for syphilis but little else except for blood groups and hemolysins. So when I had the privilege of visiting and working in developing countries I often “felt at home” as I had been through similar struggles in the development of our own services and had a great deal of empathy for the people I met and their problems.

My own experience had been in helping to formulate a blood service in two less developed countries. While working in Papua New Guinea I had the opportunity of visiting many countries in Asia and that interest continued even when I eventually returned to New Zealand to head a major transfusion service. This experience meant I saw blood services at varying stages of development, from the very basic to the quite sophisticated. With great interest I was able to follow the impressive development of the blood services of Japan, Taiwan, Hong Kong, Singapore and Korea, powerhouses in the forging of centralized and well-organized systems.

The transfer of assistance from somewhat “developed” countries to “developing” countries took some time to develop in South Asia. But it did eventually occur and my own government through an aid program took up the challenge of assisting various centers in peripheral Thailand. It worked closely with the Thai Red Cross Society and with University authorities to set up new systems and to facilitate training of national transfusion staff by holding technical workshops in Thailand and sending medical and technical staff to work for short periods in New Zealand. There was also some input into the Malaysian blood service and assistance with their preparation of blood typing antiserum. The Australian Red Cross took a great interest in Nepal and I recall in the early ‘70s preparing a report for them on the blood service there and taking a picture of the commercial donors waiting outside the donor rooms for the next accident to happen! [Figure 1]

**Figure 1:**
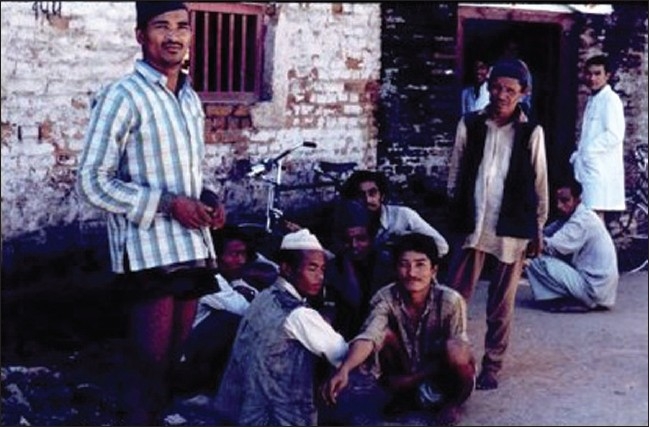
Commercial blood donors waiting in Kathmandu hospital for the next request for blood (1970s)

The Nepalese Red Cross later received the expertise of an expatriate Medical Director (a New Zealander) who set up systems that have lasted to this day. There was also input from other countries.

Good progress was made in Indonesia which despite its stage of economic development had managed to put into operation a centrally organized blood service, greatly assisted by good medical leadership. Later this was unfortunately decentralized. Malaysia was early in its blood transfusion development and the policies put in action have resulted in an effective service. The Philippines has been hampered by economic and political factors including the problems of commercial blood donors and private blood banks. The work in Viet Nam has been very slow to develop although some bright spots have emerged but the conflicts of the past, political considerations and financial considerations has not made progress easy. The regional meeting of the ISBT in Hanoi marked a new era for Viet Nam in transfusion matters. Laos, Cambodia and Myanmar (Burma) have partly tackled their problems of blood transfusion but much remains to be done although input from countries such as Japan has been welcomed.

Nepal has done well with a national blood service meeting most needs. Bangladesh has still a long way to go with only a basic service in operation but with some very dedicated blood transfusion personnel doing good work and anxious to develop further despite the problems of finance, population and lack of coordination between centers. Sri Lanka has come a great distance since my first visit there in the early ‘70s at which time the service was not developed to any degree. This has now changed and although some commercial donors still remain, blood supply has greatly improved. Huge problems still face blood services in Pakistan but there is an evident will to improve the situation and this is very apparent in some centers. However real progress will require determined government policy.

India has a patchwork of good and bad blood services with some reaching the best standards of transfusion practice. During my many visits to India, I have seen much evidence of the progress made. It is a long distance from the blood filters I saw in the ‘70s in Kerala, India [Figure 2]

**Figure 2:**
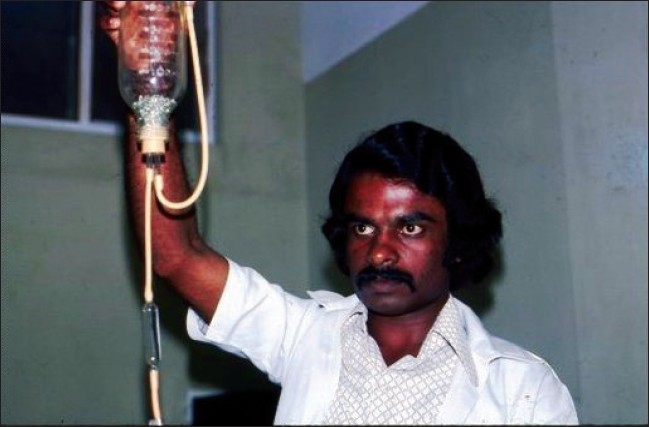
Blood transfusion equipment used in Trivandrum in the early 1970s. Note the glass bead filter and rubber tubing

At that time the blood transfusion service was the poor child of the pathology department in Trivandrum, sparsely equipped and poorly staffed. I recall spending a whole evening with the Minister of Health in Kerala trying to persuade him to develop the blood transfusion service in the State. Perhaps it worked as now the services there are reaching improved standards.

The importance of leadership in blood transfusion science and medicine is well seen in a large country like India where there are countries within a country. Based on such leadership, excellent blood services have for example, been created in Ahmedabad, Delhi, Kolkata, Chandigarh and Chennai. Strong leadership coupled with good training has greatly assisted the development of such centers despite the restrictions of finance. People with vision, insight and enthusiasm are needed if a blood service is to progress and such people should be treasured, encouraged and honored.

The input of international meetings from organizations such as the International Society of Blood Transfusion (ISBT) and the Red Cross/ Red Crescent Society should be recognized. Such events can catalyze action not only of blood service staff but also of governments. The ISBT has made sure that some of its meetings have been held in countries that could benefit from such exposure e.g. meetings in Hanoi, Bangkok, Macau and Delhi. The Red Cross Societies of Thailand and Japan have held five excellent symposia in Bangkok, between 1995-2008 and no doubt there are many other seminars less well publicized. International meetings are also a help to national blood organizations by drawing government attention to national needs, encouraging staff to undertake research into their transfusion problems and strengthening the local blood transfusion organization. This in turn has a real role in education and policy formation. Some centers in India have run their own conferences for local workers and even in some cases for international visitors e.g. the International Colloquium on Blood Donor Motivation organized by the West Bengal Association of Voluntary Blood donors. Such initiatives are very valuable with often an improvement in the morale and standards of the blood service. National Blood Transfusion organizations such as the Indian Society of Blood Transfusion and Immunohaematology (ISBTI) provide an excellent forum for discussing local problems and updating workers on contemporary transfusion policies. Every country should have a national organization and it is good to see that various South Asian countries have now evolved in this direction.

But to return to South Asia, with its great population. Some important lessons can be learnt from the development pains of selected blood services. For instance in Hong Kong, in the early ‘70s the needs of the hospitals for blood donors were mainly met by expatriates living in Hong Kong. It required a massive change in orientation to change this policy until today virtually all donors are of Chinese origin. The prevailing attitudes of the population had to be changed, no easy task especially when blood donation was presented as a voluntary altruistic action, not an easy concept for all Chinese to accept. It required excellent leadership from the donor organizers and the Medical Director, coupled with a clever and expensive publicity campaign, teaching in schools, and an emphasis on other areas of education. Experience in this service has benefited China as a whole where there is now developing a much more realistic attitude towards voluntary non-remunerated blood donation. Many Chinese citizens are accepting the challenge of becoming truly voluntary blood donors, and we can envisage that before many more years the blood services of China will be both adequate and have an ever-increasing army of safe blood donors. The myths surrounding blood donation in Oriental populations are being removed as is well shown not only in Hong Kong but also Macau, Taiwan, South Korea and elsewhere in South Asia where the voluntary donor rate is now very high.

Unfortunately, the problems of an overall shortage of blood donors in many countries of South Asia still remain. Demand for blood commonly exceeds supply and although the percentage of non-paid donors has increased, there is still the problem of a blood supply system that is inadequate. Prof Robert Beale of Australia described it so well many years ago, when he said there was “an epidemic of empty blood bank shelves in Asia.” Although the situation has improved there is still a long way to go before the shelves will be full.

There is no doubt that many South Asian countries still rely on “replacement” donors i.e. donors recruited by the family for a patient. Although some of these donors may well be from the family, more often than not they are often friends or other contacts. Not uncommonly, the family pays donors and thus professional donors still have their ways to make money from blood despite the efforts of blood services to screen them out of the system. A little bit of dishonesty gets them through the systems and because of the need to get blood the family does not ask too many questions. Although this is a shortsighted and potentially dangerous situation for the patient, it still occurs and underlines the problems of a replacement donor system. The only way to get a reliable donor service is to remove the onus for obtaining donors from the patient to a properly funded voluntary blood donor recruitment organization. Fortunately, this is starting to occur in most South Asian countries but at a rate that is often too slow.

There is little doubt, from worldwide experience, that people who are motivated to give blood for altruistic reasons help to build up a safe blood supply. But voluntary non-remunerated donors do not magically appear without effort. It requires a country or an organization to develop a strategy to appeal to the basic goodwill motivation of the population served and provide them with the reasons why they should donate blood. I have a great belief in the common humanity of all peoples, and it is my experience in many countries that most people will respond to a human need if it is presented in an acceptable way. Herein lies the problem; how to appeal to people who may have greatly varying levels of education and finance, who may have differing religions and ethnic obligations, and who are often also strongly influenced by myths and commercial pressures. In my opinion, a very successful homegrown approach has been the one developed by the West Bengal Association of Voluntary Donors, which has resulted in 85% of donors in West Bengal being voluntary. Non-paid volunteers of many backgrounds, who give of their time, energy and finance to provide the education, and also the strategy, to take the donor bed as close to the donor as possible, run the program. A wide range of listener-friendly entertaining education programs has been evolved and the involvement of the volunteers is nothing short of inspiring. The program is run with an exceedingly small budget and demonstrates what can be done with visionary leadership. Voluntary blood donation has now become the culture and the concept of volunteers recruiting volunteers has been very successful. All the literature I have seen from this program has been eminently sensible, the ideas easily transferable to other countries, and the results in producing voluntary blood donors very successful.[3]

Another alternative is a concerted effort to ensure the concepts of blood donation are taught in all schools and this has worked well in Hong Kong. Harnessing the efforts of local community organizations such as religious bodies, and service organizations including Rotary and Lion Clubs has proved successful in some areas. Not enough is done in South Asian countries to develop these concepts partly from restraints of finance and poor leadership but also because insufficient attention is paid to the importance of ensuring that a high standard of motivated public relations personnel are recruited into blood services. Sometimes it is too easy to spend finance on specialized and attractive laboratory equipment rather than on where the real needs are i.e. a lack of blood donors. In my experience donor organizer staff of this kind are usually under-paid.

There are statistics on the percentage of blood donors that are voluntary but the figures for each country need to be interpreted with care, as some data represent wishful thinking rather than hard information. So often the voluntary donors are really replacement donors or may mainly come from organizations where there is a fair degree of coercion to donate blood e.g. uniformed services (army and police) and institutions. The truly voluntary non-remunerated altruistic blood donor is still a rare bird and the regular donor even rarer in many countries of South Asia. There is a need to obtain better blood statistics based on more rigid and reliable data as otherwise blood services may be obscuring problems they really have. South Asian countries would be well advised to define what they would consider to be a reasonable incentive or recognition program for blood donors. This may well be different from that promulgated by the ISBT which although applying to well developed countries may not be entirely appropriate for countries with a financially poorer population and where a gift is expected to be replaced by a gift. A good example is the provision of a substantial meal. Is this the reason why the donor comes? If so, can such a donor be truly categorized as a voluntary donor? There is a challenge here for South Asian countries to be quite clear in their statistics by developing accurate criteria. Too often the rewards offered to attract ‘voluntary’ donors are substantial and by themselves are often enough to attract blood donors who attend to receive these rather than for any altruistic purpose. South Asian countries need to be aware of this problem and work towards developing a panel of donors who give for the ‘right’ reasons.

Quality Assurance in transfusion science and medicine was almost unknown in South Asian countries until relatively recently. But now it is an integral part of the programs of numerous transfusion services although its application to the donor service is still in its infancy. Further evolution of the concepts is required to ensure that the highest standards of safety and staff performance are achieved. This requires directed specialized training of staff members in quality matters, but this is an area that is generally neglected.

Can we be happy with the evolution of blood transfusion activities in South Asia? There is certainly no need to be depressed about them but there is still a long way to go before all the countries of the region can be assured of a safe, sufficient and reliable blood supply. Poorly functional governments, financial restrictions for the health services, lack of adequate regulations relating to blood and often lack of trained experienced personnel, hamper the development of blood services in some countries. But the main deficiency is often an absence of a clear vision by leaders, both political and at the level of the blood service. Nevertheless, progress has been generally good, and can be expected to continue along with increasing affluence in many countries. But the pressing need is still to produce sufficient voluntary blood donors to meet clinical needs and this should be the main emphasis of all South Asian countries. It is to be noted, according to WHO, only 54 countries of the world have achieved 100 percent voluntary donation and only a few of them are in South Asia.[4] There is much work still to be done and considerable finance should be poured in this direction, ideas of various countries harnessed, and education programs on blood donation extended in an effort to produce populations willing, eager and able to help in the lifesaving activities of blood services. Personally, I have few doubts that this can be achieved in the region but it will require a determined and often co-operative effort between countries to share information and expertise in the common interest.
